# Peripheral artery disease and exertional leg symptoms in diabetes patients in Ghana

**DOI:** 10.1186/s12872-016-0247-x

**Published:** 2016-04-19

**Authors:** Kwame Yeboah, Peter Puplampu, Joana Ainuson, Josephine Akpalu, Ben Gyan, Albert G. B. Amoah

**Affiliations:** Department of Physiology, School of Biomedical and Allied Health Sciences, University of Ghana, P. O. Box KB 143, Accra, Ghana; Department of Medicine & Therapeutics, School of Medicine & Dentistry, University of Ghana, Accra, Ghana; Department of Dietetics, School of Biomedical and Allied Health Sciences, University of Ghana, Accra, Ghana; Noguchi Memorial Institute of Medical Research, University of Ghana, Accra, Ghana; National Diabetes Management & Research Centre, Korle-Bu Teaching Hospital, Accra, Ghana

**Keywords:** Diabetes, Peripheral arterial disease, Ankle brachial index, Exertional leg symptoms, Ghana

## Abstract

**Background:**

Peripheral arterial disease (PAD) is a major health problem in diabetes patients in high-income countries, but the PAD burden in sub-Saharan Africa is largely undetermined. We studied the prevalence of PAD and exertional leg symptoms in diabetes (DM) patients in a tertiary hospital in Ghana.

**Methods:**

In a case control study design, 485 DM and 330 non-diabetes participants were recruited. PAD was diagnosed as Ankle Brachial Index (ABI) < 0.9. Edinburgh Claudication Questionnaire (ECQ) was used to assess exertional leg symptoms.

**Results:**

The overall prevalence of classical intermittent claudication was 10.3 % and ABI-diagnosed PAD was 26.7 %, with 3.5 % of the participants having both classic intermittent claudication and ABI-diagnosed PAD. The prevalence of exertional leg symptoms were similar in diabetes patients with and without PAD. In non-diabetes participants, intermittent claudication and rest pain were higher in PAD patients than in non-PAD participants. In multivariable logistic regression, intermittent claudication [OR (95 % CI), 3.39 (1.14 – 8.1), *p* < 0.05] and rest pain [4.3 (1.58 – 9.67), *p* < 0.001] were independently associated with PAD in non-diabetes group, and rest pain [1.71 (1.13 – 2.17), *p* < 0.05] was associated with PAD in all participants.

**Conclusions:**

There is high burden of PAD and exertional leg pains in DM patients in Ghana. PAD is expressed as intermittent claudication and rest pain in non-diabetes individuals.

**Electronic supplementary material:**

The online version of this article (doi:10.1186/s12872-016-0247-x) contains supplementary material, which is available to authorized users.

## Background

Peripheral Arterial Disease (PAD) is third leading cause of atherothrombotic macrovascular disease worldwide after coronary heart disease and stroke [1]. PAD is a common manifestation of systemic atherosclerosis, with prevalence ranging from 4 % in healthy adult population to 29 % in diabetes patients screened at outpatient clinics [2]. In sub-Saharan Africa, the burden of PAD is expected to increase, driven not only by increased life expectancy, but exposure to atherothrombotic risk factors as well [3]. Diabetes and hypertension, major chronic diseases with high PAD risk, affect 3 % [4] and 28 % [5] of Ghanaian population respectively, yet the burden of PAD in such high risk population is largely undetermined.

PAD is often diagnosed in low-resourced population using clinical history of intermittent claudication. Reduced perfusion of leg muscles (mainly calf, occasionally thigh or buttock) secondary to atherosclerotic narrowing of peripheral arteries, aggravated by exercise, leads to pain that typically subsides within 10 min of rest [6]. The Edinburgh claudication questionnaire (ECQ) contains standardized items that measure a spectrum of symptoms associated with typical pain experienced by a patients with atherosclerotic narrowing of the leg arteries [7]. However, most PAD patients are asymptomatic and clinical signs may appear at later stages of the disease [2]. PAD can be objectively diagnosed using a simple, highly reproducible, noninvasive hemodynamic test called ankle-brachial index (ABI). In addition to its diagnostic value, ABI can also be used to assess generalized atherosclerosis [8]. Previous studies have shown that low ABI (≤0.90) has specificity greater than 98 % for the diagnosis of PAD and a specificity of 92 % for the prediction of coronary heart disease and stroke [9]. In asymptomatic adults with no prior history of CVD, low ABI indicates subclinical atherosclerosis that predicts all-cause mortality and cardiovascular morbidity and death [8].

In this study, we used ECQ to investigate the burden of exertional leg symptoms in diabetes (DM) patients and non-diabetes controls, and the relationship between exertional leg symptoms and PAD assessed as ABI < 0.9. We hypothesize that, compared to non-diabetes individuals, DM patients will have higher prevalence of PAD, and there is no difference in symptomatic expression of PAD between diabetes and non-diabetes participants.

## Methods

**Fig. 1:**
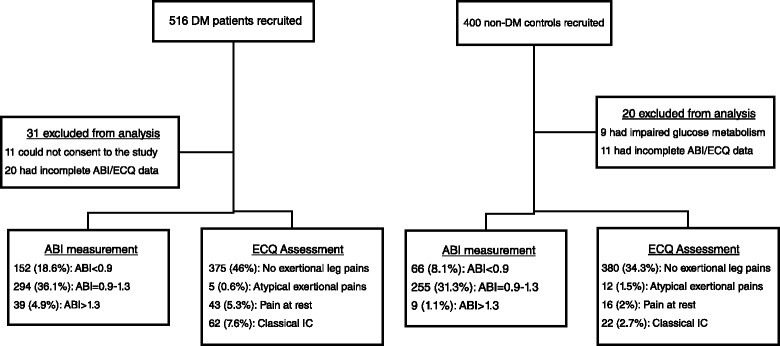
Flow chart of subjects’ recruitment

The study was case control design National Diabetes Management and Research Centre, Korle-Bu Teaching Hospital in Accra, Ghana, from June 2009 to May 2010. The centre is Ghana’s main referral clinic and operates ambulatory diabetes services and research. The cases were already diagnosed DM patients, recruited by systematic sampling, as every 3^rd^ patient vising the centre. The controls were recruited afterwards and matched with the cases by gender and age-decade. The controls were non-diabetes volunteers, with normal fasting glucose (<6.9 mmol/l) and post-glucose load plasma glucose (<7.2 mmol/l), recruited by invitation from the communities around the hospitals. Out of 1000 volunteers (600 DM and 400 non-diabetes) invited, 866 (516 DM and 350 non-diabetes) consented to participate in the study. In the final analysis, 31 diabetes (11 refused consent and 20 had incomplete ABI measurement) and 20 non-diabetes participants (9 had impaired glucose metabolism and 11 had incomplete ABI) were excluded (Fig. 1). Ethical approval for this study was obtained from the University of Ghana Medical School Ethical and Protocol Review Committee (Protocol ID number: MS-Et/M.2 – P.4.10/2009-2010) and all participants gave written informed consent after the procedures involved in the study were thoroughly explained to them.

A structured questionnaire was administered to all the participants to collect information on age, gender, education, employment status, duration of DM, DM medication, pre-existing hypertension, smoking and alcohol status. Second-hand smoking was assessed as living smoking relative or co-worker. Hypertension was defined as subjects with BP ≥ 140/90 mmHg and/or on antihypertensive medication.

Leg symptoms of patients were assessed using the ECQ (Additional file 1: Table S1), and categorized based on previous work by Criqui et al. [10]. To blind investigators of the presence or absence of PAD [11], ECQ was administered before ABI measurement. Patients were categorized based on the following definitions of leg symptoms:

1. No exertional leg pain, defined as the absence of exertional leg pain, numbness, or discomfort.
2. Atypical exertional leg pain, defined as
  - exertional calf symptoms that do not begin at rest but are otherwise not consistent with Rose intermittent claudication or
  - exertional leg symptoms that do not begin at rest and do not include the calves.
3. Pain at rest: exertional leg symptoms that also begin at rest; and
4. Classical intermittent claudication: exertional calf symptoms that do not begin at rest, worsen when walking uphill or hurrying, and resolve within 10 min of rest.

Weight, height, waist and hip girth were measured using standard protocol. [12] Briefly, body weight was determined on twice using an electronic scale (Seca 770) following due calibration (precision ± 0.1 kg), with the patient wearing light clothing with shoes removed. Height was also measured with a portable system (Seca 222) with the patient shoeless in the upright position. Body mass index (BMI) was calculated as weight (kg) divided by height squared (m^2^). Waist circumference was measured with non-elastic tape measure at the upper border of the iliac crest, parallel to the floor without compressing the skin.

Ankle and brachial pressures were measured with the patient in the supine position after at least 5 min rest. Firstly, the systolic brachial blood pressures (BP) were measured with 8 MHz Doppler (LifeDop, Summit Doppler) in both arms, after which the systolic ankle BPs of the dorsalis pedis and posterior tibial arteries were measured at the level of the malleoli with a Doppler. The ankle brachial index was calculated for each leg by dividing the highest systolic ankle pressure by the highest brachial systolic pressure [13]. The study participants were categorised into those with PAD (ABI < 0.9), normal (0.9 ≥ ABI < 1.3) and stiff arteries (ABI ≥ 1.3).

The data was analyzed using IBM SPSS Version 20. Differences in mean values of different ABI groups were assessed using ANOVA and distribution of categorical variables with *χ*^2^ test. After filtering out individuals with partially incompressible artery (ABI > 1.3), binary logistic regression model was used to determine independent clinical factors and leg symptoms associated with low ABI, compared to normal ABI. *p* < 0.05 was considered statistically significant.

## Results

**Fig. 2:**
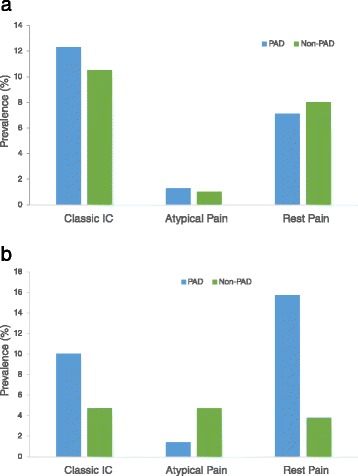
**a** Age- and gender-adjusted prevalence of exertional leg pain in diabetes patients by PAD status. **b** Age- and gender-adjusted prevalence of exertional leg pain in non-diabetes participants by PAD status

DM patients were older, with higher rate of hypertension and alcohol intake than non-diabetes controls. Also, DM patients has higher mean levels of BMI, heart rate, systolic, diastolic, mean and pulse blood pressures. The overall prevalence of ABI-diagnosed PAD was 26.7 %, with 6 % of the participants having stiff, partially incompressible artery (ABI > 1.3); more in DM patients (5.1 % vs. 0.9 %, *p* < 0.001) than non-diabetes participants. ABI categorization was associated with DM status, yet no difference in the mean ABI was observed between DM and non-diabetes controls (Table 1). Exertional leg symptoms were associated with DM status, with diabetes patients having higher age and gender adjusted prevalence of classical intermittent claudication (6.6 % vs 3.6 %, *p* < 0.05) and pain at rest (8.1 % vs 1.7 %, *p* < 0.01), but less atypical exertional pain (0.9 % vs 2.7 %, *p* < 0.01), than non-diabetes patients. In diabetes patients, age and gender adjusted prevalence of exertional leg symptoms were similar between PAD and non-PAD participants (Fig. 2a). However, in non-diabetes participants, PAD patients had higher age and gender adjusted prevalence of intermittent claudication and pain at rest tahn non-PAD participants (Fig. 2b). In all participants, 3.5 % had both PAD and classical intermittent claudication, higher in DM patients (2.6 % vs 0.9 %, *p* < 0.05) than non-diabetes participants. Compared to other leg symptoms groups, patients with intermittent claudication were older, with higher proportion of females, diabetes and hypertensive patients, and had higher systolic, diastolic and mean BPs (Additional file 2: Table S2).

**Table 1:** General characteristics of study participants by diabetes status

	All	Diabetes	Non-diabetes	*p*
	Participants	Patients	controls	
Age, yrs	54.6 ± 10.5	56.4 ± 10.4	51.9 ± 10.2	0.01
Gender (male), n (%)	368 (45.1)	212 (26)	156 (19.1)	0.79
Duration of diabetes, yrs		7.1 ± 6.2		
Hypertension, n (%)	397 (48.7)	323 (39.6)	74 (9.1)	<0.001
BMI, kg/m^2^	27.9 ± 7.8	29 ± 8.7	26.3 ± 5.8	<0.001
Height, cm	163 ± 11	162 ± 13	164 ± 8	0.038
Waist girth, cm	94 ± 24	95 ± 12	93 ± 34	0.442
Waist-hip ratio	0.93 ± 0.21	0.94 ± 0.07	0.91 ± 0.31	0.061
Systolic BP, mmHg	135 ± 26	144 ± 24	121 ± 22	<0.001
Diastolic BP, mmHg	80 ± 13	84 ± 13	74 ± 10	<0.001
Pulse BP, mmHg	55 ± 19	60 ± 18	48 ± 18	<0.001
Mean BP, mmHg	98 ± 15	104 ± 15	90 ± 11	<0.001
Heart rate, bpm	80 ± 13	82 ± 13	76 ± 12	<0.001
Smoking, n (%)				0.034
Current	36 (4.4)	10 (1.2)	26 (3.2)	
Former	139 (17.1)	101 (12.4)	38 (4.7)	
Never	630 (77.3)	364 (44.7)	266 (32.6)	
Second-hand smoking	120 (14.7)	73 (8.9)	47 (5.8)	0.078
Alcohol, n (%)	223 (27.4)	114 (14)	109 (13.4)	0.002
Educational level				0.344
Up to Elementary school	493 (60.5)	301 (36.9)	192 (23.6)	
Higher than elementary	322 (39.5)	185 (22.7)	137 (16.8)	
Employment				0.043
Unemployed	319 (39.2)	206 (25.3)	113 (13.9)	
Part-time employment	49 (6)	27 (3.3)	22 (2.7)	
Full-time employment	447 (54.8)	255 (31.3)	192 (23.5)	
Diabetes medication, n (%)				0.012
Oral hypoglycaemics		315 (38.7)		
Insulin		68 (8.3)		
Insulin & oral hypoglycaemics		102 (12.5)		
ABI (mean ± SD)	0.99 ± 0.22	0.93 ± 0.24	1.02 ± 0.17	0.115
Low (<0.9), n (%)	218 (26.7)	152 (18.6)	66 (8.1)	
Normal (0.9–1.3), n (%)	549 (67.4)	294 (36.1)	255 (31.3)	0.001
High (<1.3), n (%)	48 (6)	39 (4.9)	9 (1.1)

**Fig. 3:**
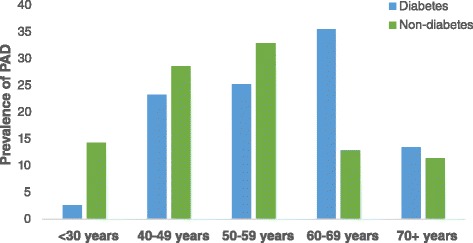
Distribution of PAD across age decades

**Fig. 4:**
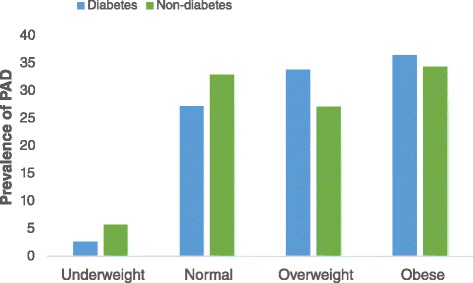
Distribution of PAD across BMI categories

Participants in various ABI categories had similar age, BMI, waist-hip ratio, systolic BP and gender distribution. PAD patients were shorter with higher diastolic BP and heart rate. Exertional leg symptoms were not associated with ABI groups (Table 2). In both genders, PAD prevalence, lower in participants younger than 40 years, increased dramatically from 4^th^ through 6^th^ decades, and reduced after 7^th^ decade of life (Fig. 3). In DM patients, except for underweight group, there was a general increase in age and gender adjusted prevalence of PAD across BMI groups. However, age and gender adjusted prevalence of PAD remained similar in non-diabetes participants across various BMI groups (Fig. 4).

**Table 2:** Characteristics of study participants by ABI categories

	PAD	Normal	Stiff Artery	*p*
	(ABI < 0.9)	(ABI = 0.9-1.3)	(ABI > 1.3)	
n (%)	213 (26.7)	536 (67.3)	48 (6)	
Age, yrs	54.1 ± 10.9	54.4 ± 10.4	56.2 ± 10.5	0.441
Gender (M/F)	87/126	262/274	20/28	0.111
Diabetes, n (%)	148 (18.6)	295 (37)	33 (4.1)	0.001
Duration, yrs	7.5 ± 6.8	6.7 ± 5.5	8.4 ± 9.1	0.168
Hypertension, n (%)	142 (18.1)	217 (27.7)	23 (2.9)	<0.001
BMI, kg/m^2^	28.9 ± 8.7	27.7 ± 7.7	26.6 ± 5.2	0.099
Height, cm	161 ± 12	163 ± 11	165 ± 7	0.021
Waist girth, cm	94 ± 14	94 ± 27	91 ± 15	0.651
Waist-hip ratio	0.91 ± 0.07	0.93 ± 0.25	0.93 ± 0.08	0.748
Systolic BP, mmHg	137 ± 24	134 ± 27	136 ± 22	0.387
Diastolic BP, mmHg	82 ± 13	79 ± 13	82 ± 18	0.04
Pulse BP, mmHg	55 ± 16	55 ± 20	55 ± 16	0.874
Mean BP, mmHg	100 ± 16	98 ± 15	98 ± 13	0.182
Heart rate, bpm	82 ± 14	79 ± 13	82 ± 18	0.013
Smoking, n (%)				
Current	8 ( 1)	29 (3.6)	0	0.727
Former	38 (4.8)	99 (12.4)	8 (1)	0.941
Never	167(21)	408(51.2)	40(5)	0.196
Second-hand smoking	37 (4.6)	77 (9.4)	6 (0.8)	0.176
Alcohol, n (%)	45 (6.1)	157 (21.4)	6 (0.8)	0.108
Leg symptoms				0.147
No exertional pain	168 (20.6)	450 (55.2)	37 (4.5)	
Classic IC	26 (3.3)	51 (6.4)	5 (0.6)	
Atypical exertional pain	7 (0.9)	9 (1.1)	1 (0.1)	
Pain at rest	16 (2)	38 (4.7)	7 (0.8)	

BMI, body mass index; BP, blood pressure; IC, intermittent claudication

Logistic multivariable regression models with and without adjustment for possible confounders were constructed to assess the independent contributions of clinical factors and exertional leg symptoms to PAD. In all participants, the unadjusted model showed that PAD was associated with increase in age, BMI, mean BP, female gender, having diabetes or hypertension, alcohol intake, second-hand smoking and use of insulin. After adjustment of confounders, diabetes and hypertension status, and the use of insulin were associated with PAD. (Additional file 3: Table S3). With respect to exertional leg symptoms, in all participants, classic intermittent claudication and pain at rest were associated with PAD in unadjusted model, but only pain at rest associated significantly with PAD in the adjusted model. Also, in non-diabetes participants symptoms of classic intermittent claudication and pain at rest were associated with PAD after adjustment of confounders (Table 3).

## Discussion

The prevalence of ABI-diagnosed PAD in this study was 26.7 % in all participants, higher in diabetes patients than non-diabetes volunteers. This fall within the reported range in most developed countries, which is 20 – 30 % [14]. In sub-Saharan Africa, data on prevalence of PAD is scanty and exhibit regional variation. For instance, community-based studies in South Africa [15] and Central African Republic [16] reported contrasting PAD prevalence in adult population to be 29.3 and 14.8 % respectively. Similarly, in diabetes patients, two studies in Uganda reported prevalence of PAD to be 24 % [17] and 39 % [18]; PAD prevalence in diabetes subgroup in this study was within this range. Since the authors used similar internationally recognized techniques in assessment and computation of ABI, other factors may be responsible for the observed disparity of PAD prevalence in sub-Saharan Africa. It has been shown that temperature [19] and altitude [20] may affect ABI measurement.

The prevalence of classical intermittent claudication in our study was 10.3 %, with 3.5 % of the participants having both PAD and classical claudication. Intermittent claudication is considered the most classic manifestation of PAD and is described as the “earliest manifestation” or “most common symptom” in PAD, yet, patients with PAD span the spectrum from asymptomatic to ischemic rest pain and threatened limb loss [21]. However, up to 90 % of persons with PAD in the general population do not have classic symptoms of intermittent claudication. In consistent with the findings of this study, the PARTNERS study reported that, in PAD patients, only 11 % had classic claudication, one-third reported no leg pain at all, whereas the remaining 55 % had atypical symptoms. [10] This highlight the limitation of using classic intermittent claudication in screening for PAD. Earlier study reported ECQ to have high sensitivity and specificity in screening for PAD [7], but data from subsequent studies reported contradictory results [15]. The findings of this study imply that, screening for PAD using ABI and ECQ may have a complimentary effect in estimating PAD burden, especially in diabetes patients [15]. Indeed, patients with intermittent claudication and low ABI are reported to have greater deterioration of the large and small arteries [22], leading to severe tissue hypoperfusion.

In this study, we found that PAD was associated differently with exertional leg symptoms in diabetes and non-diabetes participants. While the prevalence of intermittent claudication and rest pain were similar in diabetes patients with and without PAD, in non-diabetes subjects, intermittent claudication and rest pain were higher in those with PAD than in non-PAD subjects (Fig. 2a & b). Also, in the multivariable regression model (Table 3) intermittent claudication and rest pain were associated with PAD in non-diabetes subjects only. Compared to non-diabetes individuals, diabetes patients have poorer lower extremity functioning, limiting their engagement in exertional activities leading to leg pains, symptomatical of PAD [23]. Ischemic damage of sensory nerves, resulting from vaso-occlusion, in PAD patients may affect the perception of exertional leg symptoms. [24] However, in diabetes patients, damage of sensory nerves from vascular-dependent and vascular-independent mechanisms obscure leg symptoms typical of PAD [25]. Therefore, in our study population, PAD was manifested as intermittent claudication and rest pain in non-diabetic participants, and asymptomatic in diabetes patients. This observation agrees with other reported studies in which PAD was asymptomatic in diabetes patients [6, 9, 24].

**Table 3:** Association between exertional leg symptoms and ABI-diagnosed PAD by of participants by their diabetes status

	Crude OR (95 % CI)	*p*	Adjusted OR* (95 % CI)	*p*
*All Participants*				
Classic IC	1.59 (1.19–2.69)	0.047	1.51 (0.87–2.61)	0.139
Atypical exertional pain	0.54 (0.15–1.89)	0.331	0.32 (0.07–1.46)	0.148
Pain at rest	1.72 (1.21–3.04)	0.036	1.71 (1.13–2.17)	0.028
*Diabetes Patients*				
Classic IC	1.18 (0.64–2.19)	0.59	1.02 (0.54–1.94)	0.569
Atypical exertional pain	1.25 (0.21–7.56)	0.811	0.49 (0.05–4.93)	0.547
Pain at rest	0.89 (0.42–1.9)	0.771	0.89 (0.4–2)	0.769
*Non-diabetes Participants*				
Classic IC	2.56 (1.07–5.92)	0.039	3.39 (1.14–8.1)	0.028
Atypical exertional pain	0.37 (0.05–2.9)	0.358	0.31 (0.03–2.48)	0.266
Pain at rest	4.91 (1.93–12.49)	0.001	4.3 (1.58–9.67)	0.004

IC, intermittent claudication; OR, odds ratio.

*Adjusted for age, gender, diabetes status (in All participants group only), insulin use (in Diabetes group only), hypertension, BMI, waist-hip ratio, mean blood pressure, smoking status, second-hand smoking and alcohol status

Except for smoking, the major risk factors for PAD (age, diabetes and hypertension) in our study was similar to that of the Framingham Offspring Study. Smoking is known to increase PAD risk, whereas cessation of smoking status with decreases risk of PAD [26, 27]. Contrary to what has been reported in literature, we did not observe any association between smoking status and PAD, possibly due to the low prevalence of current and former smokers in our study population. The low prevalence of smoking in our study is similar to that reported in the Demographic Health Survey in Ghana, in which smoking prevalence was reported to be 4.3 % [28, 29]. However, given that the low prevalence of smoking, a major risk factor for PAD, contributing factors for low ABI should be investigated in sub-Saharan Africa population.

In this study, we found the prevalence of PAD higher in female participants than male counterparts. This is consistent with the results from MESA [30] and ARIC [31] studies, where the prevalence of low ABI were higher in females. Further studies, probing into the intrinsic characteristics of arterial constitution of males and females may help to explain this observation. Contrary to most reported studies, classic intermittent claudication in our study was more prevalent in females than males. In both in original [32] and offspring Framingham Studies [33], majority of incidence of intermittent claudication occurred in females. However, their study population was mainly Caucasians with high smoking rate compared to our study population. The prevalence of PAD increased across age decades, similar to other studies, except from 7^th^ decade onwards, where prevalence of PAD decreased dramatically. This may be attributed, in concert with the life expectancy in Ghana (60 years), to increased mortality in Ghanaian population after 6^th^ decade. Consistent with global burden of PAD [1], more female participants had PAD than their male counterparts. Further studies may investigate whether atherosclerotic plaques is responsible for low ABI in females or females have intrinsically low ankle BPs which may be misdiagnosed as PAD.

The findings of this study showed that the prevalence of falsely elevated ABI (ABI > 1.3) is 6 %, more in diabetes patients than non-diabetes participants. This prevalence of elevated ABI in this study was lower than what was reported in Finish study [34] and Strong Heart Study [35]. Elevated ABI indicates medial arterial calcinosis, which precludes accurate ABI assessment. In comparison to the results from digital subtraction angiography, 62 % of patients with elevated ABI has PAD, defined as more than 50 % narrowing of the arterial lumen in any arterial segment [34]. Elevated ABI is reported to confer high mortality risk from all-causes and CVDs, similar to those with low ABI, highlighting the U-shape relationship between ABI and CVD outcomes [35].

### Limitations of the study

The study was conducted in tertiary referral clinic, and therefore, urban–rural dynamics of ABI and leg symptoms were not investigated; this affects the generalizability of the results to the entire population. The data of this study was collected cross-sectionally with no organ damage assessed. Therefore, the predictive value of ABI and exertional leg symptoms cannot be inferred from the results. In addition, non-diabetic controls in this study were conveniently sampled, and hence, their burden of PAD may not reflect the actual burden in the population. Peripheral sensory neuropathy, which is an important link between low ABI and perception of leg symptoms, was not assessed in the study. Also, no humoural biomarker was assayed to study the underlining pathophysiological mechanism driving the high burden of PAD in our study population. Future studies may utilize longitudinal community-based studies, with object organ damage assessment and relevant humoural markers, to investigate the utility and predictive value of ABI in sub-Saharan African population.

## Conclusion

The findings of the present study have shown high burden of PAD and exertion leg pains in DM patient in urban Ghana irrespective of low smoking rate. PAD was associated with intermittent claudication and rest pain in non-diabetes individuals.

### Ethics, consent and permissions

The study was conducted in accordance with the Declaration of Helsinki and ethical approval was granted by the University of Ghana Medical School Ethical and Protocol Review Committee (Protocol ID number: MS-Et/M.2 – P.4.10/2012-2013). All participants gave written informed consent after thorough explanation of the procedures involved.

### Availability of data and materials

The dataset supporting the conclusions of this article is included within the article as Additional files.

## Additional files

Additional file 1: Table S1. Edinburgh claudication questionnaire. (DOCX 13 kb) Additional file 2: Table S2. Characteristics of study participants by leg symptoms. (DOCX 13 kb) Additional file 3: Table S3. Association of clinical factors and PAD. (DOCX 13 kb)

## Abbreviations

ABI: ankle-brachial index
CVDs: cardiovascular diseases
ECQ: Edinburgh intermittent claudication questionnaire
PAD: peripheral arterial disease
T2DM: type 2 diabetes
