# Supplementary material for: Peripheral artery disease and exertional leg symptoms in diabetes patients in Ghana
Source: BMC Cardiovasc Disord. 2016 Apr 19;16:68. doi: 10.1186/s12872-016-0247-x (PMC4837554; doi:10.1186/s12872-016-0247-x)
Supplement: Additional file 1: Table S1. — Edinburgh claudication questionnaire. (DOCX 13 kb) [file 12872_2016_247_MOESM1_ESM.docx]

Additional file 1: Table S1. Edinburgh Claudication Questionnaire

| 1. Do you get pain/discomfort on either leg on walking?  Yes  No 2. Does the pain ever begin when you are standing or sitting?  Yes  No 3. In what part of your leg do you feel it? (mark ‘X’ on the diagram below)   Pain include calf/calves  Pain does not include calf/calves   - 1. If pain does not include calf, is it felt anywhere? Please specify………..………………  1. Do you get it when you walk uphill or hurry?  Yes  No   Never hurry or walk uphill   1. Do you get it when you walk in an ordinary pace or level?  Yes  No 2. Does the pain disappear while you are walking?  Yes No 3. What do you do if you get it while you are walking?  Stop and slow down   Carry on   1. What happens to it if you stand still?  Relieved  Not relieved 2. How soon?  10 minutes or less  More than 10 minutes |
| --- |
