# Supplementary material for: Peripheral artery disease and exertional leg symptoms in diabetes patients in Ghana
Source: BMC Cardiovasc Disord. 2016 Apr 19;16:68. doi: 10.1186/s12872-016-0247-x (PMC4837554; doi:10.1186/s12872-016-0247-x)
Supplement: Additional file 2: Table S2. — Characteristics of study participants by leg symptoms. (DOCX 13 kb) [file 12872_2016_247_MOESM2_ESM.docx]

Additional file 2: Table S2 Characteristics of Study Participants by Leg Symptoms

|  | No Leg Pain | Classical IC | Atypical Pain | Rest Pain | p |
| --- | --- | --- | --- | --- | --- |
| **Age, yrs** | **54.1±10.3** | **57.1±11.5** | **56±9.8** | **56.3±11.3** | **0.046** |
| **Female, n (%)** | **343 (41.8)** | **78 (6.4)** | **17 (1.3)** | **60 (4.4)** | **0.045** |
| **Diabetes, n (%)** | **387 (47.5)** | **66 (8.1)** | **5 (0.6)** | **38 (4.6)** | **0.002** |
| **Hypertension, n (%)** | **305 (37.8)** | **50 (6.2)** | **4 (0.5)** | **34 (4.2)** | **0.001** |
| BMI, kg/m^2^ | 27.6±7.7 | 28.9±7.5 | 27.1±3.9 | 29.9±9.9 | 0.105 |
| Height, cm | 163±11 | 162±11 | 160±6 | 162±11 | 0.268 |
| Waist girth, cm | 93±20 | 100±49 | 90±10 | 97±18 | 0.063 |
| Waist-hip ratio | 0.92±0.17 | 0.97±0.43 | 0.89±0.06 | 0.94±0.09 | 0.17 |
| **Systolic BP, mmHg** | **134±26** | **144±26** | **124±18** | **132±23** | **0.003** |
| **Diastolic BP, mmHg** | **80±13** | **84±13** | **72±9** | **79±13** | **0.002** |
| Pulse BP, mmHg | 55±19 | 60±21 | 52±16 | 53±15 | 0.091 |
| **Mean BP, mmHg** | **98±15** | **104±16** | **89±10** | **97±15** | **0.001** |
| Heart rate, bpm | 80±13 | 80±12 | 79±14 | 78±12 | 0.793 |
| Current smoking, n (%) | 33 (4) | 1 (0.1) | 2 (0.2) | 1 (0.1) | 0.213 |
| Second-hand smoking | 96 (11.8) | 17 (2.1) | 0 | 7 (0.9) | 0.091 |
| Alcohol, n (%) | 188 (22.9) | 15 (1.8) | 3 (0.4) | 19 (2.3) | 0.213 |

IC, intermittent claudication; BMI, body mass index; BP, blood pressure
