# Supplementary material for: Peripheral artery disease and exertional leg symptoms in diabetes patients in Ghana
Source: BMC Cardiovasc Disord. 2016 Apr 19;16:68. doi: 10.1186/s12872-016-0247-x (PMC4837554; doi:10.1186/s12872-016-0247-x)
Supplement: Additional file 3: Table S3. — Association of clinical factors and PAD. (DOCX 13 kb) [file 12872_2016_247_MOESM3_ESM.docx]

Additional file 3: Table S3 Association of Clinical Factors and PAD

|  | | | Crude OR (95% CI) |  |  | Adjusted OR (95% CI)* |  |
| --- | --- | --- | --- | --- | --- | --- | --- |
| Age | | | **1.03 (1.01 – 1.04)** | **0.001** |  | - |  |
|  | Age per 5-year | | **1.14 (1.06 – 1.23)** | **0.001** |  | - |  |
| Gender (female) | | | **1.35 (1.17 – 1.65)** | **0.041** |  | - |  |
| Diabetes | | | **1.89 (1.34 – 2.67)** | **<0.001** |  | **1.61 (1.09 – 2.39)** | **0.018** |
|  | Duration | | 1.03 (0.99 – 1.06) | 0.124 |  |  |  |
| Hypertension | | | **2.69 (1.94 – 3.73)** | **<0.001** |  | **3.24 (2.1 – 5)** | **<0.001** |
| BMI, kg/m^2^ | | | **1.02 (1.01 – 1.04)** | **0.021** |  | - |  |
|  | BMI per SD | | **1.2 (1.03 – 1.39)** | **0.021** |  | - |  |
| Waist girth, cm | | | 1.02 (0.99 – 1.09) | 0.425 |  | - |  |
| Waist-hip ratio | | | 1.08 (0.53 – 2.2) | 0.829 |  | - |  |
| Systolic BP, mmHg | | | 1.01 (0.99 – 1.05) | 0.109 |  | - |  |
| Diastolic BP, mmHg | | | 1.01 (0.99 – 1.02) | 0.12 |  | - |  |
| Pulse BP, mmHg | | | 1.01 (1 – 1.02) | 0.064 |  | - |  |
| Mean BP, mmHg | | | **1.01 (1.01 – 1.02)** | **0.035** |  | - |  |
|  | Mean BP per 10 mmHg | | **1.12 (1.01 – 1.24)** | **0.035** |  | - |  |
| Heart rate, bpm | | | 1.01 (1 – 1.02) | 0.05 |  | - |  |
| Smoking, (Ref: Never smoked) | | | |  |  |  |  |
|  | | Current | 0.62 (0.28 – 1.38) | 0.243 |  | - |  |
|  | | Former | 0.99 (0.66 – 1.5) | 0.698 |  | - |  |
| Second-hand smoking | | | 0.98 (0.63 – 1.58) | 0.91 |  | - |  |
| Alcohol | | | **1.14 (1.08 – 1.21)** | **0.048** |  | 1.02 (0.69 – 1.49) | 0.397 |
| Educational level (Ref: Higher than elementary) | | | | |  |  |  |
|  | | Up to Elementary school | **1.38 (1.09 – 1.83)** | **0.047** |  | 1.3 (0.91 – 1.86) | 0.153 |
| Employment (Ref: Unemployed) | | | |  |  |  |  |
|  | | Part-time employment | 0.63 (0.31 – 1.28) | 0.203 |  | 0.73 (0.35 – 1.51) | 0.393 |
|  | | Full-time employment | **0.62 (0.45 – 0.85)** | **0.004** |  | 0.73 (0.51 – 1.06) | 0.068 |
| Insulin use | | | **1.5 (1.08 – 2.61)** | **0.043** |  | **1.76 (1.04 – 3.15)** | **0.047** |

BMI, body mass index; BP, blood pressure.

*Adjusted for age, gender, BMI, waist-hip ratio, waist circumference, mean blood pressure, heart rate, smoking status & second-hand smoking.
